# The Nursery Elf (Poetry)

**Published:** 1881-07

**Authors:** 


### THE NURSERY ELF.

Dear little feet, how you wander and wander,
Little twin truants so fleet!
Dear little head, how you ponder and ponder,
Over the things that you meet!
Dear little tongue, how you chatter and chatter
Over your innocent joys!
Oh, but the house is alive with your clatter—
Shaking, indeed, with your noise!

Can't you be quiet a moment, sweet rover?
Is there no end to your fun?
Soon the old "sand man" will sprinkle you over,
Then the day's frolic is done.

Come to my arms, for the daylight is dying.
Closer the dark shadows creep;
Come, like a bird that is weary of flying;
Come, let me sing you to sleep.

